# Italian version of the Occupational Depression Inventory: Validity, reliability, and associations with health, economic, and work-life characteristics

**DOI:** 10.3389/fpsyt.2022.1061293

**Published:** 2022-12-22

**Authors:** Renzo Bianchi, Caterina Fiorilli, Giacomo Angelini, Nicoletta Dozio, Carlo Palazzi, Gloria Palazzi, Benedetto Vitiello, Irvin Sam Schonfeld

**Affiliations:** ^1^Department of Psychology, Norwegian University of Science and Technology (NTNU), Trondheim, Norway; ^2^Department of Social Sciences (Communication, Education and Psychology), LUMSA University, Rome, Italy; ^3^Azienda Socio-Sanitaria Territoriale (ASST) della Brianza, Vimercate, Italy; ^4^Faculty of Natural and Mathematical Sciences, King's College London, London, United Kingdom; ^5^LexisNexis, London, United Kingdom; ^6^Department of Public Health and Pediatrics, University of Turin, Turin, Italy; ^7^Department of Mental Health, Johns Hopkins University School of Public Health, Baltimore, MD, United States; ^8^Department of Psychology, The City College and the Graduate Center of the City University of New York, New York, NY, United States

**Keywords:** job-related distress, factor analysis, Mokken scale analysis, occupational health, sick leave, economic stress, workplace violence, burnout

## Abstract

**Background:**

The Occupational Depression Inventory (ODI) reflects a new approach to job-related distress centered on work-attributed depressive symptoms. The instrument was developed with reference to the characterization of major depression found in the *Diagnostic and statistical manual of mental disorders*, fifth edition. The ODI has been validated in English, French, and Spanish. This study (a) investigated the psychometric and structural properties of the ODI's Italian version and (b) inquired into the nomological network of occupational depression.

**Methods:**

A convenience sample of 963 employed individuals was recruited in Italy (69.9% female; mean age = 40.433). We notably relied on exploratory structural equation modeling bifactor analysis, common-practice confirmatory factor analysis, and Mokken scale analysis to examine our dataset.

**Results:**

Our analyses indicated that the Italian version of the ODI meets the requirements for essential unidimensionality, thus justifying the use of the instrument's total score. The ODI's reliability was excellent. Measurement invariance held across sexes, age groups, and occupations. Occupational depression was negatively associated with general wellbeing and positively associated with a 12-month history of depressive disorder, current antidepressant intake, 12-month sick leave, 6-month physical assault at work, 6-month verbal abuse at work, lack of money for leisure activities, and financial strain in the household.

**Conclusions:**

The ODI's Italian version exhibits robust psychometric and structural properties, suggesting that the instrument can be fruitfully used for addressing job-related distress in Italian-speaking populations. Furthermore, the present study relates occupational depression to important health, economic, and work-life characteristics, including past depressive episodes, antidepressant medication, sickness-related absenteeism, workplace violence, and economic stress.

## 1. Background

Job-related distress constitutes a research hotspot in occupational health science (1–3). However, fierce debates surround the conceptualization and measurement of the phenomenon (4–7). It is in this context that the Occupational Depression Inventory (ODI) was developed (8). The ODI is part of a renewed approach to job-related distress. The instrument is designed to assess work-attributed depressive symptoms and identify likely cases of occupational depression. The ODI thus approaches job-related distress both dimensionally (continuum-based approach) and categorically (diagnostic approach). In contrast to the items of “classical” depression scales, the items of the ODI incorporate causal attributions to work (e.g., “My work was so stressful that I could not enjoy the things that I usually like doing”). The use of causal attributions has been commonplace in psychological science, for instance, to identify sources of stress in the general population (9). The ODI focuses on the nine core symptoms of major depression (10) and includes a subsidiary item assessing turnover intention in relation to job-related distress. Research on job-related distress has been slowed down by a lack of robust, well-defined indicators (6, 7, 11). The ODI was created to improve occupational health specialists' ability to address job-related distress (12).

The ODI has been validated in English, French, and Spanish thus far (8, 13–17). The instrument has been employed in the USA, Australia, New Zealand, South Africa, Spain, Switzerland, and France. The ODI has consistently exhibited robust psychometric and structural properties, as revealed by in-depth validity and reliability analyses—including exploratory structural equation modeling (ESEM) bifactor analysis and Mokken scale analysis (18–20). The measure has shown a combination of convergent validity and discriminant validity when examined against classical, attribution-free depression scales (8, 13, 16). In terms of its criterion validity and nomological network, occupational depression has been linked to a variety of job-related and context-free variables, including work engagement, job satisfaction, social support at work, job autonomy, trait anxiety, general health status, and objective cognitive performance (8, 13–16). Furthermore, the instrument has served to clarify the controversial issue of burnout-depression overlap (17, 21). ODI-based research has carried further the notion that burnout symptoms are part of a depressive syndrome and do not reflect a unique or distinct entity.

The ODI responds to many shortcomings affecting popular indicators of job-related distress such as burnout (5, 22, 23). In contrast to the Maslach Burnout Inventory (MBI)—the measure of reference for assessing burnout symptoms, the ODI (a) adopts both a dimensional and a categorical approach to job-related distress, (b) allows for prevalence estimation, (c) assesses suicidal ideation—a marker of severe job-related distress, (d) benefits from solid clinical and theoretical foundations, (e) exhibits sound psychometric and structural properties, and (f) shows well-aligned conceptualization and measurement (12, 23). The ODI is also briefer and easier to use than the MBI. The MBI comprises a higher number of items (16 or 22, depending on the version) and produces three separate scores that are difficult to coordinate (e.g., to obtain a global index of burnout). Finally, while the MBI is a proprietary test, the ODI is available to researchers and practitioners at no cost.

The objective of the present study was twofold. First, we aimed to validate the ODI in the Italian language. The Italian version of the ODI has not been tested thus far. It is important to determine whether its psychometric and structural properties are satisfactory. We addressed this first objective by relying on sophisticated statistical techniques, such as ESEM bifactor analysis (18). ESEM bifactor analysis is a valuable resource for investigating an instrument's factorial structure and ascertaining whether a measure is “unidimensional enough” to support the use of observed total scores (20). Second, we aimed to inquire further into the nomological network of occupational depression. Because the ODI was released recently, our knowledge of the predictors, correlates, and outcomes of occupational depression is still limited. We addressed this second objective by focusing on health, economic, and work-life indicators thought to be particularly relevant to occupational depression. We examined the associations of occupational depression with a history of depressive disorder, antidepressant intake, sick leave, job promotion, physical assault at work, verbal abuse at work, lack of money for leisure activities, financial strain in the household, and general wellbeing. Overall, we submitted the ODI to a stringent examination, consistent with recommendations for closer scrutiny of psychological scales' validity and reliability (24, 25).

## 2. Methods

### 2.1. Sample of interest and participant recruitment

A convenience sample of 963 Italian employees [69.9% female (*n* = 673)] was surveyed in 2022. Participants were employed in a variety of occupational sectors although a large proportion of participants were schoolteachers (*n* = 456). Participants' mean age was 40.433 (*SD* = 10.611). The sample was recruited from training events addressing an occupational stress prevention program.

Respondents took part in the study on a voluntary basis. Participation involved no compensation. Respondents were guaranteed full confidentiality, in compliance with privacy rights described in current Italian law (Law Decree DL-196/2003). Informed consent was obtained from all subjects. The study met the ethical standards of the institutional review board of LUMSA University (Rome, Italy; Prot. N. 6/2021).

### 2.2. Measures of interest

#### 2.2.1. ODI

The ODI, initially developed by Bianchi and Schonfeld (8), was our principal measure of interest. The ODI comprises nine core symptom items referencing the diagnostic symptoms for major depression found in the *Diagnostic and statistical manual of mental disorders*, fifth edition (DSM-5) (10). The ODI assesses the symptoms of interest within a 2-week time window. Each symptom item is rated from 0 for “never or almost never” to 3 for “nearly every day.” The ODI additionally includes a question related to turnover intention, associated with three response options (“yes,” “no,” and “I don't know”). The ODI is accompanied by instructions that invite respondents to reflect on the sources of their symptoms before answering (e.g., work-unrelated sources). This precaution aims to deter hasty attributions of symptoms to work.

The ODI is intended to be used either dimensionally, based on the scale's total score, or categorically, based on a dedicated diagnostic algorithm (8). The ODI's diagnostic algorithm does *not* consist in a cutoff score that would demarcate clinically relevant levels of symptoms from subclinical levels of symptoms. The ODI's diagnostic algorithm is founded on the DSM-5's diagnostic criteria for major depression and takes into account, for instance, the primacy of anhedonia and depressed mood in depression's symptomatology (8, 10). The ODI's diagnostic algorithm allows investigators to identify likely cases of occupational depression; the diagnosis is considered *provisional* because it is based on self-report rather than a clinician-driven interview (26). The diagnostic algorithm is detailed in Supplementary material 1.

We used a back-translation method to translate the ODI into Italian (27). First, the English version was translated into Italian by two native Italian speakers fluent in English. Second, the Italian version was translated back into English by a bilingual Italian and English speaker. Neither the English-to-Italian nor the Italian-to-English translators were familiar with the measure before taking part in the translation process. Third, we compared the English version derived from the back-translation with the original English version. We did not identify any problematic discrepancies. The items of the ODI are available in Italian and English in Table 1. The full Italian version of the ODI, which includes the instructions to respondents, is provided in Supplementary material 1.

**Table 1:** Italian version of the items of the Occupational Depression Inventory (ODI).

**Symptoms**	**Items**
Anhedonia	Il mio lavoro era così stressante che non riuscivo ad apprezzare le attività che di solito mi piacciono *My work was so stressful that I could not enjoy the things that I usually like doing*
Depressed mood	Mi sono sentito depresso/a a causa del mio lavoro *I felt depressed because of my job*
Sleep alterations	Lo stress del lavoro mi ha causato problemi di sonno (ho avuto difficoltà ad addormentarmi o a dormire, oppure ho dormito molto più del solito) *The stress of my job caused me to have sleep problems (I had difficulties falling asleep or staying asleep, or I slept much more than usual)*
Fatigue/loss of energy	Mi sono sentito/a esausto/a a causa del mio lavoro *I felt exhausted because of my work*
Appetite alterations	Ho sentito che il mio appetito era disturbato a causa dello stress del mio lavoro (ho perso il mio appetito, o al contrario, ho mangiato troppo) *I felt my appetite was disturbed because of the stress of my job (I lost my appetite, or the opposite, I ate too much)*
Feelings of worthlessness	La mia esperienza al lavoro mi ha fatto sentire come un/a fallito/a *My experience at work made me feel like a failure*
Cognitive impairment	Il mio lavoro mi ha stressato così tanto che facevo fatica a concentrarmi su quello che stavo facendo (ad esempio leggere un articolo di giornale) o a pensare chiaramente (ad esempio prendere decisioni) *My job stressed me so much that I had trouble focusing on what I was doing (e.g., reading a newspaper article) or thinking clearly (e.g., to make decisions)*
Psychomotor alterations	A causa dello stress da lavoro, mi sono sentito/a irrequieto/a e incapace di star fermo/a, o al contrario, mi sono sentito/a rallentato/a—ad esempio nel modo in cui mi muovevo o parlavo *As a result of job stress, I felt restless, or the opposite, noticeably slowed down—for example, in the way I moved or spoke*
Suicidal ideation	Ho pensato che preferirei essere morto/a piuttosto che continuare in questo lavoro *I thought that I*'*d rather be dead than continue in this job*
Turnover intention (SQ)	Se hai avvertito almeno qualcuno dei problemi menzionati qui sopra, questi problemi ti hanno portato a considerare di lasciare il tuo attuale lavoro o la tua posizione? *If you have encountered at least some of the problems mentioned above, do these problems lead you to consider leaving your current job or position?*

The full ODI form (including the instructions to respondents) is available in Italian in Supplementary material 1, together with an SPSS syntax implementing the provisional diagnosis algorithm of the ODI.

SQ, subsidiary question.

#### 2.2.2. History of depressive disorder and antidepressant intake

Participants were asked to indicate whether they had been diagnosed with a depressive disorder by a health professional over the past year. Response options were “yes,” “no,” and “I'm not sure.” In addition, participants were asked about whether they were currently under antidepressant medication. Again, response options were “yes,” “no,” and “I'm not sure.”

#### 2.2.3. Sick leave and job promotion

Participants answered yes/no questions about whether they had been (a) on sick leave and (b) promoted in their job (as reflected in higher status and/or income) at some point over the past year.

#### 2.2.4. Physical assault and verbal abuse at work

Participants were asked to indicate whether they had been (a) physically assaulted and (b) verbally abused in the context of their work over the past 6 months. Response options were “yes,” “no,” and “I'm not sure.”

#### 2.2.5. Lack of money for leisure activities and financial strain

Lack of money for leisure activities was assessed with the following yes/no item: “Do you have enough money to pursue your hobbies and passions?” (28). Financial strain was assessed with the following item: “How would you describe the money situation in your household right now?” (29). Response options were: “comfortable with extra” (1); “enough but no extra” (2); “have to cut back” (3); “cannot make ends meet” (4).

#### 2.2.6. General wellbeing

We assessed general wellbeing with the Flourishing Scale (FS) (30, 31). The FS comprises eight items rated from 1 for “strongly disagree” to 7 for “strongly agree.” A sample item is: “I am competent and capable in the activities that are important to me.” In this study, the FS exhibited a Cronbach's alpha of 0.869 and a McDonald's omega of 0.894.

### 2.3. Data analysis

We ran our factor analyses with Mplus 8.6 (32). We first examined the ODI's factorial structure within an ESEM bifactor analytic framework (18). We relied on a partially specified target rotation (PSTR). Compared to “classical” confirmatory factor analysis (CFA), the PSTR does not fix nontarget loadings at 0; instead, nontarget loadings are “encouraged” to get as close to 0 as possible, allowing factorial complexity to be modeled. Consistent with Bianchi and Schonfeld's (8) findings on the ODI's factorial structure, we extracted two specific factors (or bifactors) in addition to the general Occupational Depression factor. The ODI's “anhedonic-somatic” items (Items 1, 3, 4, 5, 7, and 8) were directed toward the first specific factor; the ODI's “dysphoric” items (Items 2, 6, and 9) were directed toward the second specific factor. We used an orthogonal PSTR—the bifactors were not allowed to correlate. We approached the ODI items as ordinal and used the weighted least squares—mean and variance adjusted—estimator. To ascertain how the general factor accounted for the common variance extracted, we computed the Explained Common Variance (ECV) statistic (20). An ECV index exceeding 0.80 is considered to signal essential unidimensionality. We relied on the omega and omega hierarchical (omegaH) coefficients to scrutinize the ODI's reliability and the general factor's correlation with the observed total scores. We further inquired into the factorial structure of the ODI using “classical” CFA. We tested a one-factor model—we set all ODI items to load on a single factor.

In a final effort to assess the ODI's dimensionality, we estimated the scale's homogeneity (or scalability) within a Mokken scale analytic framework (19, 33). We conducted the analysis with the Mokken package version 3.0.6 (34) in R version 4.0.3 (35). Homogeneity refers to the extent to which a scale's items hierarchically align on a single dimension. The hierarchy concerns *item difficulty*, i.e., the likelihood for an item to be endorsed by respondents. In the context of psychopathology items, item difficulty is equivalent to symptom severity. In the ODI, we expect, for instance, the fatigue/loss of energy item to be less “difficult” (i.e., more frequently endorsed) than the suicidal ideation item because suicidal ideation represents a much more severe symptom than fatigue/loss of energy. Homogeneity is indexed by *H* coefficients. As per commonly applied rules of thumb (36), a scale's homogeneity is regarded as weak if 0.30 ≤ *H* < 0.40, moderate if 0.40 ≤ *H* < 0.50, and strong if *H* ≥ 0.50; a scale-level *H* coefficient below 0.30 suggests that the scale of interest cannot be regarded as unidimensional. Pairwise *H* coefficients should be >0. Item-level *H* coefficients should be >0.30. In addition to computing *H* coefficients, we relied on the automated item selection procedure (AISP), a method for evaluating scale formation. The AISP enables us to identify subscales and deviating or unscalable items (37). We computed Cronbach's alpha, Guttman's lambda-2, and the Molenaar-Sijtsma statistic as additional reliability indicators.

We investigated the measurement invariance of a unidimensional model across sexes (male/female), age groups (based on a tercile split), and occupations (teachers/other professions) focusing on: (a) configural invariance—the equivalence at the level of model forms; (b) metric invariance—the equivalence at the level of factor loadings; and (c) scalar invariance—the equivalence at the level of item thresholds (38). We relied on conservative standards for flagging measurement invariance violations: 0.005 for ΔRMSEA and ΔSRMR; and −0.005 for ΔCFI and ΔTLI (38, 39).

We examined the criterion validity and nomological network of the ODI based on Pearson and Spearman correlations as well as Welch's analysis of variance (ANOVA). Welch's ANOVA is a robust test of equality of means that allows investigators to cope with homoscedasticity violations.

## 3. Results

We found the distribution of ODI mean scores to be positively skewed (skew = 1.181, standard error = 0.079), which is unsurprising given our focus on a nonclinical sample. ODI mean scores ranged from 0.000 to 2.778. Scores on each of the symptom items ranged from 0.000 to 3.000. Of our 963 participants, 75.5% (*n* = 727) scored between 0.000 and 0.999, 21.4% (*n* = 206) scored between 1.000 and 1.999, and 3.1% (*n* = 30) scored between 2.000 and 3.000. We identified 1.8% of the participants (*n* = 17) as likely cases of occupational depression. An examination of the ODI's turnover intention item revealed that 27.1% of the participants (*n* = 261) were considering leaving their current job or position.

### 3.1. Factor-analytic findings

**Figure 1:**
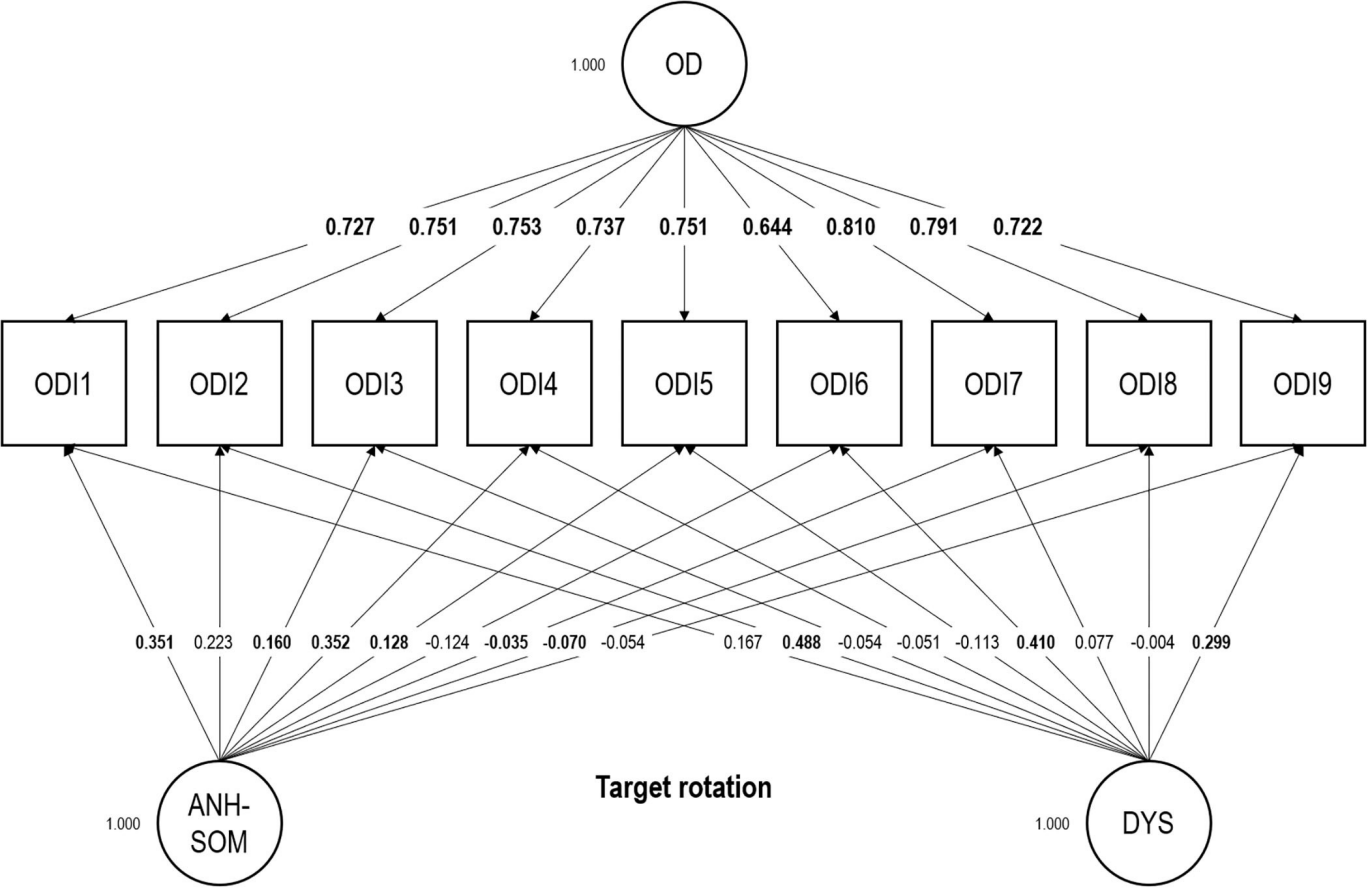
Exploratory structural equation modeling bifactor analysis of the Occupational Depression Inventory—factor loadings. Target loadings are bolded. OD, general Occupational Depression factor; ANH-SOM, anhedonic-somatic bifactor; DYS, dysphoric bifactor. *N* = 963 (no missing values); ODI1, anhedonia; ODI2, depressed mood; ODI3, sleep alterations; ODI4, fatigue/loss of energy; ODI5, appetite alterations; ODI6, feelings of worthlessness; ODI7, cognitive impairment; ODI8, psychomotor alterations; ODI9, suicidal ideation.

Regarding the ODI, our ESEM bifactor analytic structure showed a satisfactory fit: RMSEA = 0.047; CFI = 0.997; TLI = 0.990; SRMR = 0.014; χ^2^(12) = 38.023. Factor loadings are displayed in Figure 1. The mean factor loading on the general factor was 0.743 (*SD* = 0.047). The ECV index indicated that the general factor accounted for 85.3% of the common variance extracted. Omega was 0.941 and OmegaH, 0.846. We found a correlation of 0.920 between the general factor and the observed total scores. Comparing OmegaH with Omega, we found that most of the reliable variance in observed total scores could be attributed to the general factor (0.846/0.941 = 0.899), assumed to reflect individual differences in occupational depression.

**Figure 2:**
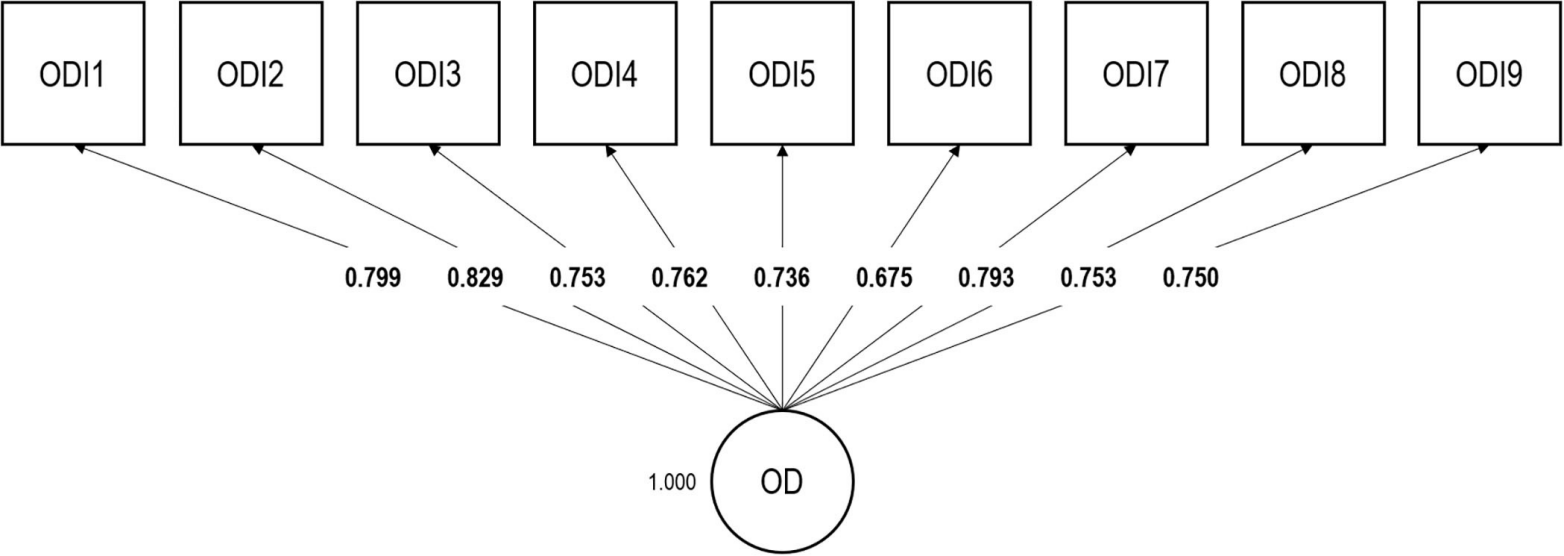
Confirmatory factor analysis of the Occupational Depression Inventory—factor loadings. OD, Occupational Depression factor. *N* = 963 (no missing values); ODI1, anhedonia; ODI2, depressed mood; ODI3, sleep alterations; ODI4, fatigue/loss of energy; ODI5, appetite alterations; ODI6, feelings of worthlessness; ODI7, cognitive impairment; ODI8, psychomotor alterations; ODI9, suicidal ideation.

Consistent with our ESEM bifactor analytic findings, a one-factor confirmatory analytic model showed an acceptable fit: RMSEA = 0.078; CFI = 0.980; TLI = 0.973; SRMR = 0.052; χ^2^(27) = 186.737. Factor loadings are displayed in Figure 2. The mean factor loading was 0.761 (*SD* = 0.044). Omega was 0.926.

A one-factor confirmatory analytic model exhibited measurement invariance across sexes, age groups, and occupations (Table 2). Regarding sexes, RMSEA never increased and TLI never decreased; CFI never decreased by more than 0.001 and, in a similar vein, SRMR never increased by more than 0.001. Regarding age groups, RMSEA never increased and TLI never decreased; CFI remained virtually identical as constraints were added; SRMR never increased by more than 0.003. Regarding occupations, RMSEA never increased by more than 0.003 and TLI never decreased by more than 0.002; CFI never decreased by more than 0.005; SRMR never increased by more than 0.002 as constraints were added.

**Table 2:** Measurement invariance analysis of the Occupational Depression Inventory.

**Measurement invariance**	***χ^2^***	**df**	**RMSEA**	**ΔRMSEA**	**CFI**	**ΔCFI**	**TLI**	**ΔTLI**	**SRMR**	**ΔSRMR**
**Sexes**										
Configural model	208.963	54	0.077	—	0.981	—	0.974	—	0.040	—
Metric model	225.489	62	0.074	−0.003	0.980	−0.001	0.977	0.003	0.041	0.001
Scalar model	212.424	79	0.059	−0.015	0.984	0.004	0.985	0.008	0.042	0.001
**Age groups** ^a^										
Configural model	239.698	81	0.078	—	0.981	—	0.975	—	0.044	—
Metric model	256.734	97	0.072	−0.006	0.981	0.000	0.979	0.004	0.046	0.002
Scalar model	285.718	129	0.062	−0.010	0.981	0.000	0.984	0.005	0.049	0.003
**Occupations**										
Configural model	224.962	54	0.081	—	0.979	—	0.972	—	0.043	—
Metric model	271.520	62	0.084	0.003	0.974	−0.005	0.970	−0.002	0.045	0.002
Scalar model	249.358	79	0.067	−0.017	0.979	0.005	0.981	0.011	0.046	0.001

N = 963 (no missing values).

RMSEA, Root Mean Square Error of Approximation; ΔRMSEA, delta (change in) RMSEA; CFI, Comparative Fit Index; ΔCFI, delta (change in) CFI; TLI, Tucker-Lewis Index; ΔTLI, delta (change in) TLI; SRMR, Standardized Root Mean Squared Residual; ΔSRMR, delta (change in) SRMR; df, degrees of freedom.

a Scores of 3 on ODI's Item 9 (suicidal ideation) were extremely rare (n = 4) and not present in all age groups; to be able to conduct the analysis across age groups, we thus recoded the scores of 3 into scores of 2.

### 3.2. Mokken scaling

Results of our Mokken scale analysis are presented in Table 3. The ODI exhibited strong homogeneity. The scale-level *H* coefficient reached 0.548 (95% confidence interval: 0.514, 0.582), with a standard error of only 0.017. The pairwise *H* coefficients largely exceeded the zero threshold and the item-level *H* coefficients were well above the 0.300 threshold. The AISP, used with increments of 0.005, signaled a single scale involving all ODI items up to a threshold of 0.475. The most frequently endorsed item was the fatigue/loss of energy item (Item 4) and the least frequently endorsed item was the suicidal ideation item (Item 9). Cronbach's alpha, Guttman's lambda-2, and the Molenaar-Sijtsma statistic had values ≥0.878 (Table 3).

**Table 3:** Homogeneity and reliability analyses of the Occupational Depression Inventory.

**Homogeneity and reliability**			
**Items**	***H**_*i*_*	**SE**	**95% CI**
ODI1: anhedonia	0.573	0.019	0.536, 0.610
ODI2: depressed mood	0.587	0.019	0.549, 0.626
ODI3: sleep alterations	0.553	0.021	0.512, 0.594
ODI4: fatigue/loss of energy	0.594	0.022	0.551, 0.636
ODI5: appetite alterations	0.519	0.022	0.477, 0.562
ODI6: feelings of worthlessness	0.475	0.028	0.420, 0.530
ODI7: cognitive impairment	0.563	0.021	0.521, 0.605
ODI8: psychomotor alterations	0.527	0.023	0.482, 0.573
ODI9: suicidal ideation	0.517	0.038	0.442, 0.592
*H_*ij*_*	Min = 0.391, Max = 0.685		
*H*	0.548	0.017	0.514, 0.582
AISP	0.475		
Cronbach's alpha	0.878		
Guttman's lambda-2	0.885		
Molenaar-Sijtsma statistic	0.887		

N = 963 (no missing values).

H, scale-level H; Hi, item-level H; H_ij_, pairwise-level; SE, standard error; 95% CI, 95% confidence interval; AISP, Automated Item Selection Procedure.

### 3.3. Criterion validity and nomological network

Occupational depression correlated negatively with general wellbeing, Pearson *r* = −0.392 (*p* < 0.001), Spearman ρ = −0.408 (*p* < 0.001), and positively with financial strain, Pearson *r* = 0.185 (*p* < 0.001), Spearman ρ = 0.176 (*p* < 0.001). The correlation between occupational depression and age was small and statistically nonsignificant, Pearson *r* = −0.036 (*p* = 0.260), Spearman ρ = −0.046 (*p* = 0.152). Descriptive statistics for these variables are available in Supplementary material 2.

Welch's ANOVA revealed positive associations of occupational depression with a 12-month history of depressive disorder, antidepressant intake, 12-month sick leave, 6-month physical assault at work, 6-month verbal abuse at work, and lack of money for leisure activities; occupational depression showed no links to participants' 12-month promotion and participants' sex (see Table 4 for a summary of the results). As per Cohen's (40) interpretation grid, the associations of occupational depression with a 12-month history of depressive disorder, antidepressant intake, 6-month physical assault at work, and 6-month verbal abuse at work were large or close to large in magnitude (Cohen's *d*s ranging from 0.724 to 1.082).

**Table 4:** Robust tests of equality of means.

**Variables**	**Groups**	***n***	**ODI mean score (*SD*)**	**Welch's ANOVA**	**Effect size**
12-month history of depressive disorder	No	885	0.619 (0.511)	*p* < 0.001	*d* = 0.751
	Yes	51	1.013 (0.728)		
Antidepressant intake	No	935	0.638 (0.524)	*p* < 0.001	*d* = 0.724
	Yes	27	1.025 (0.837)		
12-month sick leave	No	849	0.628 (0.523)	*p* = 0.003	*d* = 0.340
	Yes	114	0.810 (0.619)		
12-month job promotion	No	765	0.650 (0.535)	*p* = 0.965	*d* = 0.004
	Yes	198	0.648 (0.552)		
6-month physical assault at work	No	916	0.631 (0.526)	*p* = 0.001	*d* = 0.810
	Yes	36	1.062 (0.674)		
6-month verbal abuse at work	No	840	0.582 (0.483)	*p* < 0.001	*d* = 1.082
	Yes	93	1.129 (0.677)		
Lack of money for leisure activities	No	615	0.573 (0.497)	*p* < 0.001	*d* = 0.514
	Yes	159	0.839 (0.589)		
Sex	Male	290	0.616 (0.536)	*p* = 0.206	*d* = 0.089
	Female	673	0.664 (0.539)		

ANOVA, analysis of variance; ODI, Occupational Depression Inventory.

Individuals who selected “I'm not sure” responses were excluded from the analyses.

## 4. Discussion

The ODI is part of a renewed approach to job-related distress. The instrument focuses on depressive symptoms that individuals ascribe to their work. The goal of our study was to (a) examine the psychometric and structural properties of the ODI's Italian version and (b) inquire into the nomological network of occupational depression. We relied on a sample of 963 employed individuals recruited in Italy. We made use of sophisticated statistical techniques, including ESEM bifactor analysis.

### 4.1. Main findings

ESEM bifactor analysis, common-practice CFA, and Mokken scale analysis consistently indicated that the ODI's Italian version meets the requirements for essential unidimensionality, thus justifying the use of the instrument's total score. Moreover, we found the ODI to exhibit high reliability on the basis of five different indicators—omega, omegaH, Cronbach's alpha, Guttman's lambda-2, and the Molenaar-Sijtsma statistic. Our findings are consistent with the results of previous ODI studies that employed the measure in its English, French, and Spanish versions (8, 13–17). Our measurement invariance analysis supports the use of the ODI for comparisons between (a) men and women, (b) individuals across adulthood, and (c) individuals from different occupational domains.

Occupational depression was negatively linked to general wellbeing and positively linked to a 12-month history of depressive disorder, current antidepressant intake, 12-month sick leave, 6-month physical assault at work, 6-month verbal abuse at work, lack of money for leisure activities, and financial strain in the household. The links that we observed were generally medium to large in size. Our findings demonstrate the criterion validity of the ODI and further illuminate the nomological network of occupational depression. Our results are consistent with findings emanating from research on job stress and antidepressant medicine (41, 42), workplace bullying and health (43), and economic stress and employee wellbeing (44, 45).

All in all, our findings are consistent with the research on the ODI in English-, French-, and Spanish-speaking samples. The validity and reliability of the ODI in those samples can be extended to Italian-speaking samples. As previously mentioned, the present study provides new information. It links the ODI to past episodes of depression, the use of antidepressants, and general wellbeing. It also connects the ODI to economic stress, sick leave, and workplace violence.

### 4.2. Study limitations

At least four limitations to this study are noteworthy. First, although our sample was relatively large (*N* = 963) and included individuals displaying various ODI scores (reflective of various levels of symptom severity), its representativeness is unclear (e.g., in terms of sex, age, and health status). As a consequence, our estimate of occupational depression's prevalence should *not* be generalized to the Italian working population. The implementation of methods such as random sampling, which promotes sample representativeness, is very costly and frequently unworkable (e.g., because the population of interest cannot be accurately circumscribed or exhaustively contacted) (46). Unsurprisingly, such methods have rarely been used in clinical and occupational health sciences.

Second, we relied exclusively on self-reported measures, within a cross-sectional design. We note, however, that several of our self-reported measures addressed “objective” events likely to be readily identified by respondents (e.g., sick leave and job promotion over the previous year). Moreover, many of our items were retrospective in nature, a characteristic that optimizes the informativeness of cross-sectional designs (47).

Third, we relied on single-item measures to assess several of our variables of interest. Although multiple-item measures are generally considered more robust, there is evidence that single-item measures represent an acceptable measurement approach for many constructs in organizational science (48).

Fourth, our study did not reexamine the overlap between burnout and depression. Fortunately, this issue has been addressed extensively in past research (4, 46, 49–52), including ODI-based research (17, 21). The advantages of relying on the construct of (occupational) depression have been discussed on many occasions (12, 22, 23, 53–55).

## 5. Conclusions

The Italian version of the ODI exhibits robust psychometric and structural properties, suggesting that the instrument can be fruitfully used by investigators interested in job-related distress. Furthermore, our findings relate occupational depression to important health, economic, and work-life characteristics, including past depressive episodes, antidepressant medication, sickness-related absenteeism, workplace violence, and economic stress. Our results dovetail with an increasing set of findings indicating that the ODI can help researchers, practitioners, and policymakers tackle the issue of job-related distress more effectively, to the benefit of individuals, organizations, and society as a whole.

## Data availability statement

The raw data supporting the conclusions of this article will be made available by the authors, without undue reservation.

## Ethics statement

The study involved human participants. The study was reviewed and approved by the institutional review board of LUMSA University (Rome, Italy; Prot. N. 6/2021). The participants provided written informed consent to take part in the study.

## Author contributions

All authors listed have made a substantial, direct, and intellectual contribution to the work and approved it for publication.
