# Supplementary material for: Italian version of the Occupational Depression Inventory: Validity, reliability, and associations with health, economic, and work-life characteristics
Source: Front Psychiatry. 2022 Dec 22;13:1061293. doi: 10.3389/fpsyt.2022.1061293 (PMC9813419; doi:10.3389/fpsyt.2022.1061293)
Supplement: Supplementary file 1 [file Data_Sheet_1.PDF]

QUESTIONARIO PER LA DEPRESSIONE OCCUPAZIONALE (QDO)

ISTRUZIONI PER LA COMPILAZIONE

Le seguenti affermazioni riguardano l'impatto che il tuo lavoro potrebbe aver avuto su di te.

Leggi ogni affermazione e indica quanto spesso hai avvertito i problemi menzionati nelle ULTIME DUE SETTIMANE. Usa la seguente scala per rispondere:

0 = mai o quasi mai

1 = solo qualche giorno

2 = più della metà dei giorni

3 = quasi tutti i giorni

Ecco un esempio:

"Mi sono sentito/a in ansia a causa del mio lavoro."

• Se NON ti sei sentito/a in ansia a causa del tuo lavoro, seleziona **0**.

• Se ti sei sentito/a in ansia per motivi che NON SONO COLLEGATI AL TUO LAVORO (problemi personali, problemi coniugali, problemi di famiglia, problemi di salute ecc.), seleziona **0** anche in questo caso.

• Se ti sei sentito/a in ansia ma non sai perché, seleziona **0**.

• Se ti è chiaro che IL TUO LAVORO è stato la causa del tuo stato d'ansia, seleziona **1**, **2** o **3** per indicare quante volte è successo.

Ora puoi completare il questionario.

QUESTIONARIO PER LA DEPRESSIONE OCCUPAZIONALE (QDP)

Nome del paziente: ……………………………………………………   Data: …………………………………………

**Indica quanto spesso hai avvertito i problemi menzionati qui sotto nelle ultime due settimane .**	*Mai o quasi mai*	*Solo qualche giorno*	*Più della metà dei giorni*	*Quasi tutti i giorni*
1. Il mio lavoro era così stressante che non riuscivo ad apprezzare le attività che di solito mi piacciono.	**0**	**1**	**2**	**3**
2. Mi sono sentito depresso/a a causa del mio lavoro.	**0**	**1**	**2**	**3**
3. Lo stress del lavoro mi ha causato problemi di sonno (ho avuto difficoltà ad addormentarmi o a dormire, oppure ho dormito molto più del solito).	**0**	**1**	**2**	**3**
4. Mi sono sentito/a esausto/a a causa del mio lavoro.	**0**	**1**	**2**	**3**
5. Ho sentito che il mio appetito era disturbato a causa dello stress del mio lavoro (ho perso il mio appetito, o al contrario, ho mangiato troppo).	**0**	**1**	**2**	**3**
6. La mia esperienza al lavoro mi ha fatto sentire come un/a fallito/a.	**0**	**1**	**2**	**3**
7. Il mio lavoro mi ha stressato così tanto che facevo fatica a concentrarmi su quello che stavo facendo (ad esempio leggere un articolo di giornale) o a pensare chiaramente (ad esempio prendere decisioni).	**0**	**1**	**2**	**3**
8. A causa dello stress da lavoro, mi sono sentito/a irrequieto/a e incapace di star fermo/a, o al contrario, mi sono sentito/a rallentato/a – ad esempio nel modo in cui mi muovevo o parlavo.	**0**	**1**	**2**	**3**
9. Ho pensato che preferirei essere morto/a piuttosto che continuare in questo lavoro.	**0**	**1**	**2**	**3**

PUNTEGGIO TOTALE: ……………………………………………………

Se hai avvertito almeno qualcuno dei problemi menzionati qui sopra, questi problemi ti hanno portato a considerare di lasciare il tuo attuale lavoro o la tua posizione?

☐ Sì ☐ No ☐ Non lo so

Occupational Depression Inventory: SPSS syntax for a provisional diagnosis of occupational depression

```
compute DEP = 0.
do if ODI1 ge 3 or ODI2 ge 3.
count DEP = ODI3 (3)
ODI4 (3)
ODI5 (3)
ODI6 (3)
ODI7 (3)
ODI8 (3)
ODI9 (1,2,3).
end if.

if ODI1 ge 3 DEP = DEP + 1.
if ODI2 ge 3 DEP = DEP + 1.

compute DIAG = 0.
if DEP ge 5 DIAG = 1.
```

Note. The nine items of the Occupational Depression Inventory are coded ODI1 to ODI9.

Items

ODI1: anhedonia

ODI2: depressed mood

ODI3: sleep alterations

ODI4: fatigue/loss of energy

ODI5: appetite alterations

ODI6: feelings of worthlessness

ODI7: cognitive impairment

ODI8: psychomotor alterations

ODI9: suicidal ideation
