# Supplementary material for: Italian version of the Occupational Depression Inventory: Validity, reliability, and associations with health, economic, and work-life characteristics
Source: Front Psychiatry. 2022 Dec 22;13:1061293. doi: 10.3389/fpsyt.2022.1061293 (PMC9813419; doi:10.3389/fpsyt.2022.1061293)
Supplement: Supplementary file 2 [file Table_1.pdf]

Supplementary Material 2. Descriptive statistics related to occupational depression, financial strain, general well-being, and age.

	Rating scale	Observed minimum	Observed maximum	*M*	*SD*	Median	Skewness (*SE* = 0.079)	Kurtosis (*SE* = 0.157)
Occupational depression	0-3	0	2.778	0.649	0.538	0.556	1.181	1.245
Financial strain*	1-4	1	4	1.916	0.781	2	0.634	0.116
General well-being	1-7	1	7.000	5.552	0.893	5.750	-1.249	2.533
Age	N/A	18	75	44.433	10.611	40.000	0.286	-0.636

Notes. *N* = 963 (no missing values). *M*: mean; *SD*: standard deviation; *SE*: standard error. *: single-item measure.
